# Acute Neuromuscular Adaptation at the Spinal Level Following Middle Cerebral Artery Occlusion-Reperfusion in the Rat

**DOI:** 10.1371/journal.pone.0089953

**Published:** 2014-02-28

**Authors:** Caroline Pin-Barre, Jérôme Laurin, Marie-Solenne Felix, Vincent Pertici, Frank Kober, Tanguy Marqueste, Valery Matarazzo, Françoise Muscatelli-Bossy, Jean-Jacques Temprado, Jeanick Brisswalter, Patrick Decherchi

**Affiliations:** 1 Aix-Marseille Université, Centre National de la Recherche Scientifique, Institut des Sciences du Mouvement, Faculté des Sciences du Sport, Marseille, France; 2 Université de Nice Sophia-Antipolis et Université du Sud Toulon-Var, Motricité Humaine Éducation Sport Santé, Nice, France; 3 Aix-Marseille Université, Institut National de la Santé et de la Recherche Médicale, Institut de Neurobiologie de la Méditerranée, Marseille, France; 4 Aix-Marseille Université, Centre National de la Recherche Scientifique, Centre de Résonance Magnétique Biologique et Médicale, Faculté de Médecine Timone, Marseille, France; University of Naples Federico II, Italy

## Abstract

The purpose of the study was to highlight the acute motor reflex adaptation and to deepen functional deficits following a middle cerebral artery occlusion-reperfusion (MCAO-r). Thirty-six Sprague-Dawley rats were included in this study. The middle cerebral artery occlusion (MCAO; 120 min) was performed on 16 rats studied at 1 and 7 days, respectively (MCAO-D1 and MCAO-D7, n = 8 for each group). The other animals were divided into 3 groups: SHAM-D1 (n = 6), SHAM-D7 (n = 6) and Control (n = 8). Rats performed 4 behavioral tests (the elevated body swing test, the beam balance test, the ladder-climbing test and the forelimb grip force) before the surgery and daily after MCAO-r. H-reflex on *triceps brachii* was measured before and after isometric exercise. Infarction size and cerebral edema were respectively assessed by histological (Cresyl violet) and MRI measurements at the same time points than H-reflex recordings. Animals with cerebral ischemia showed persistent functional deficits during the first week post-MCAO-r. H-reflex was not decreased in response to isometric exercise one day after the cerebral ischemia contrary to the other groups. The motor reflex regulation was recovered 7 days post-MCAO-r. This result reflects an acute sensorimotor adaptation at the spinal level after MCAO-r.

## Introduction

To date, stroke rehabilitation and pharmacological treatments cannot ensure a complete functional recovery after stroke [*1*], [*2*]. Therefore, stroke survivors frequently preserve, among others, major chronic sensory and motor dysfunctions. To assess treatments and discover new ones, the understanding of the neural adaptation mechanisms underlying the functional impairments seems to be essential [*3*]. The neural mechanisms induced by stroke refer to both neuronal damages and consecutive neuromuscular adaptations around and below the lesion site (including the neural network reorganization both in the spinal cord and in the spared cerebral structures). However, these mechanisms remain poorly understood.

To explore the underlying neural adaptation, several experimental models of focal cerebral ischemia were commonly used in rats. The “Middle Cerebral Artery Occlusion-reperfusion” (MCAO-r) is one of the most frequently used cerebral ischemia method to study the neuronal death mechanisms and the severity of functional outcomes by the use of several behavioral tests [*4*]–[*6*]. There is a growing consensus that the effective evaluation of therapies in the rat MCAO-r model requires combination of histological measurements and behavioral tests. Indeed, studies showed functional disturbances after ischemia despite normal or near-normal histology [*7*]. Moreover, some others demonstrated that pharmacological treatments appeared to be ineffective based on measurement of infarct volume but were found to significantly improve functional outcome [*8*], [*9*].

However, two major limitations hampered the understanding of the pathophysiology of MCAO-r. On one hand, although the overall behavioral tests enabled to detect disorders of the motor control [*5*], [10]–[*12*], they could not determine spinal and supraspinal mechanisms responsible of the specific neural adaptation to MCAO-r. Moreover, some functional outcomes concerning the forelimbs stay controversial and need to be deepened. It is the case of the effect of cerebral ischemia on the grip force of the forelimbs, which is an important functional disorder after stroke. Indeed, several authors showed a decline in force production whereas other rat studies indicated an increase of grip force following MCAO-r [*13*], [*14*]. On the other hand, the amount of necrotic tissue in the primary infarct site is only considered to be one factor influencing functional recovery [*5*], [10]–[*12*]. As behavioral tests, histological measurements could not inform on the underlying neuromuscular alterations affecting the contralateral limbs to the cerebral ischemia [*15*].

The motor reflex regulation at the spinal level is recognized as an important aspect of the motor control and may be modulated by cerebral ischemia, despite the fact that controversial results remain for the upper-extremity control in human studies [*16*]–[*19*]. The Hoffmann reflex (H-reflex) has often been used in preclinical human and animal studies to explore the spinal sensorimotor adaptation during and/or after skeletal muscle activity [*20*], [*21*]. The H-reflex pathway is well-known not to be exclusively affected by afferent inputs from agonist/antagonist muscles and joints, but also, by descending motor pathways during movement [*22*]. However, no evidence indicates that muscular exercise-induced spinal sensorimotor adaptation could be changed by the MCAO-r in rats.

Hence, the present study was designed to assess the acute adaptation of the spinal motor reflex during the 1^st^ week following MCAO-r. For that purpose, the spinal sensorimotor adaptation was assessed by measuring the response of *triceps brachii* H-reflex before and after isometric exercise on this muscle. It is important to indicate that physical exercise can be used as a diagnostic tool revealing new changes in neuromuscular mechanisms adaptations that cannot be observed in resting condition. Indeed, physical exercise, as prolonged isometric contraction, was known to activate the excitatory and/or inhibitory mechanisms responsible of the motor unit recruitment [*23*]. In addition, this study aimed at deepening functional deficits knowledge following MCAO-r that needs to be clarified. Reproducibility and lesion size at the right hemisphere level were quantified by histological and magnetic resonance imagery analysis.

In our study, the different measurements were performed during the first week post-MCAO-r because the lesion size mainly evolved during this acute period in rat model [*24*]. It was demonstrated that the peak of cerebral damages occurred 24 hours after the lesion [*24*]–[*26*]. Several studies also indicated that the cerebral lesion size began to be stable from the 7^th^ days [*27*]–[*29*]. In addition, the processes of cerebral neuronal plasticity were initiated during the first 24 hours and were increasingly active during several days post-cerebral ischemia [*29*], [*30*]. It is noteworthy to add that the first day post-MCAO-r was considered to be an optimal time point to start pharmacological treatment or functional rehabilitation due to the opportune plasticity processes [*3*], [29], [31], [*32*].

## Materials and Methods

### 1. Animals

Fifty adult male Sprague Dawley rats, weighing exclusively between 250–270 g (Centre d'Élevage Roger JANVIER, Le Genest Saint Isle, France), were singly housed in smooth-bottomed plastic cages in a colony room maintained on a 12-h light/dark cycle. Food and water were available *ad libitum*. Animals weighting more than 270 g were excluded from the study. The room temperature was maintained at 22°C. The weight of the rats was daily controlled. In order to accustom the animals to the laboratory environment, an acclimation period of 2 weeks was allowed before the initiation of the experiment.

#### Ethics statement

Anesthesia and surgical procedures were performed according to the French law on animal care guidelines and the Animal Care Committees of *Aix-Marseille Université* (AMU) and *Centre National de la Recherche Scientifique* (CNRS) approved our protocols. Furthermore, experiments were performed following the recommendations provided in the *Guide for Care and Use of Laboratory Animals* (U.S. Department of Health and Human Services, National Institutes of Health) and in accordance with the European Community council directive of 24 November 1986 (86/609/EEC). No sign of screech, prostration and hyperactivity were observed through the experiment.

Among the 50 rats, 30 of them underwent MCAO-r surgery and the 16 survivors were equally distributed into the MCAO-D1 and the MCAO-D7 groups, as indicated below. Thus, the survival rate after MCAO-r surgery, comprising the rats of both MCAO-D1 and MCAO-D7 groups, was 53.3%. Consequently, the remaining 36 animals were randomly assigned to the following 5 groups: 1) Control (n = 8), 2) MCAO-D1 group (n = 8) in which electrophysiological, histological and magnetic resonance imagery (MRI) recordings were realized 1 day after MCAO-r, 3) MCAO-D7 group (n = 8) in which electrophysiological, histological and MRI recordings were realized 7 days after MCAO-r, 4) SHAM-D1 group (n = 6) in which electrophysiological, histological and MRI recordings were realized 1 day after the surgery without the MCAO-r step and 5) SHAM-D7 group (n = 6) in which electrophysiological, histological and MRI recordings were realized 7 days after the surgery without the MCAO-r step.

### 2. Surgical procedure: cerebral ischemia by MCAO-r

It is important to note that reproducibility of the induced deleterious effects by the surgery could be optimized by rigorously controlling temperature, weight, age, sex, type of used rat and ischemia duration [*33*]. Central temperature was maintained at about 37–38°C with a homeothermic blanket (Homeothermic Blanket Control Unit, K01345CE, Holliston, MA, USA) driven by a rectal thermal probe.

Briefly, as described in previous studies [*34*], [*35*], anaesthesia was induced with 5% isoflurane and maintained with 2.5-2% isoflurane through a facemask (Anesteo, Villetelle, France), supplemented with oxygen. A 0.2 ml injection of 0.5% bupivacaine was subcutaneously performed along the prospective incision site. A 4 cm ventral midline incision was performed and the right external, internal, and common carotid arteries (ECA, ICA, CCA) were exposed without damaging the vagus nerve and its collaterals. After a partial arteriotomy on ECA with micro-scissors, a 4-0 monofilament nylon suture (total length: 3 cm; silicon-coated tip length and diameter: 5 mm and 0.39±0.02 mm respectively; MCAO suture PK10, 40-333PK10, Redland, CA, USA) was inserted into the ICA via the ECA and approximately pushed 20 mm away from the carotid bifurcation. Blood flow was thus blocked at the MCA origin. After 120 min of occlusion, the monofilament was carefully removed and the ICA was permanently closed by electrocoagulation to prevent bleeding. Finally, the skin was sutured and animals returned in individual cages.

### 3. Behavioral tests

**Figure 1:**
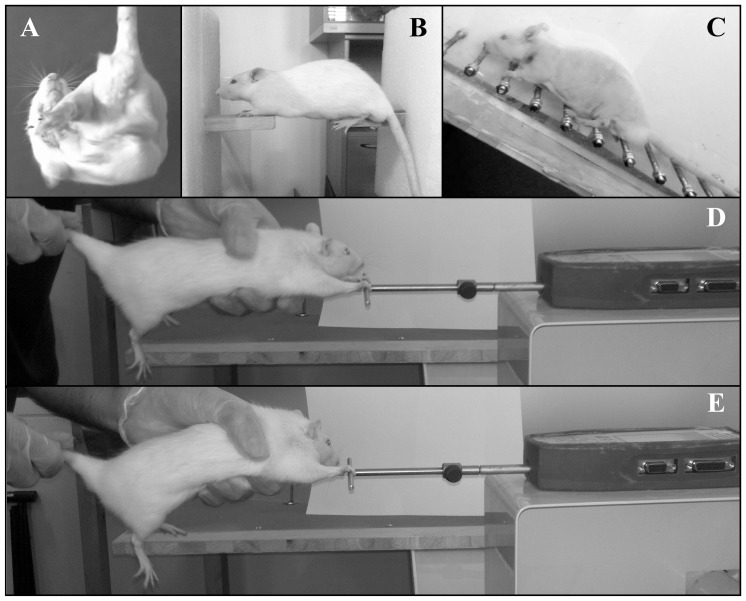
Illustration of the behavioral tests. **A**. The elevated body swing, **B**. The balance test on 2 wooden beams, **C**. The ladder-climbing test, **D**. The grip force test with both forelimbs and **E**. The grip force test with only one forelimb (in this example, the right forelimb).

The severity of functional outcomes following MCAO-r was assessed by the use of 4 behavioral tests. It also allowed us to test the reliability of the cerebral ischemia on functional outcomes between animals ***(*** ***Figure 1*** ***)***.

#### Elevated body swing test (EBST)

The EBST reflects the lateralization of the lesion (asymmetrical motor behavior) ***(*** ***Figure 1A*** ***)***. The animal was held approximately 1 cm from the base of its tail [*36*]. It was then elevated above the surface in the vertical axis. A swing was recorded whenever the animal moved its head out of the vertical axis to either the left or the right side (more than 10 degrees). Before attempting another swing, the animal was momentarily placed back on the ground of his cage. Ten swings were performed for each recording session. The overall number of swings made to the left side was divided by the overall number of swings made to both sides. For example, if the animal performed 7 swings on the left side and 3 swings on the right side, the score was 7/10 (left swing number/total swing number = 0.7)

#### Beam balance test

The beam balance task is a widely used technique to assess deficits of the vestibulomotor function in different traumatic situations [37]. The beam balance test in static position is considered not to be sensitive enough to detect vestibulomotor alteration after cerebral ischemia [*10*], [*37*]. In the present study, this task was modified to increase its sensitivity in order to detect balance deficits. The forelimbs and the hindlimbs were respectively positioned on distinct narrow wooden beams (diameter: 28 mm; space length: 5 cm) ***(*** ***Figure 1B*** ***)***. Therefore, rats could only balance themselves using their 4 paws excluding any other body part support. The time to fall off the rod was recorded. The animals performed 2 trials with a maximum time fixed at 60 s. Finally, the total recording time was calculated by adding the time of the two trials.

#### Ladder-climbing test

The ladder-climbing test is used to evaluate the sensorimotor capacities to correctly grip the rung while rats climbed up a inclined ladder ***(*** ***Figure 1C*** ***)*** [*38*]. In our study, the ladder was inclined to a 45° angle (length: 100 cm; width scale: 13 cm). The animals walked across the inclined ladder with rungs of equal spacing (1 cm). The rats performed 4–5 trials per testing session. The video analysis of the left paw grip was recorded with a 100-Hz acquisition frequency using SimiMotion software (Unterschleissheim, Germany) associated with a numerical camcorder (MV 830i; Canon, Courbevoie, France). The number of grips of the left paw was counted (20 steps minimum). Then, the number of mistakes, misses or slips, per crossing was taken into account. If the grip on the rung was correct, the score was 2 points. If the rung was completely missed by the left forepaw, the score was 0 point. If the paw slipped on the rung or the grip was incomplete, the score was 1 point. Moreover, if the left paw touched intermediate rungs during the swing phase, the score was also 1 point. For each testing session, the successful score was normalized with the maximal score, depending on the number of performed steps. Results were expressed as percentage of PRE-values. For example, if the animal performed 40 steps (maximal score = 80) and the score including mistakes was 45, the result was 45/80 (successful score = 56%).

#### Forelimb grip force

The grip force exerted by both forepaws together and by each forepaw was measured by using a grip force tester [Grip Strength Tester (GST) bio-GT3, Bioseb, Vitrolles, France] ***(*** ***Figure 1D*** ***)***. The same experimenter performed all the grip force measurements to obtain more reproducible and reliable data. To standardize the assessment of grip force, the rat was held by the base of the tail above the bar and was then moved down until its forelimbs grasped the middle of the bar. While the body and the lower limbs were horizontally maintained, the experimenter pulled the rat following the axis of the sensor (horizontally) until the grasp was released. The trial ended when the rat released the bar (or failed to establish grip on the GST bar). During each trial, the maximum force developed by the rat was obtained just before grip was released. An individual testing trial lasted 5–7 s. The time interval between each trial was fixed to 1 min to avoid fatigue accumulation. A positive grip was scored when the digits extended and then flexed upon contacting the bar followed by the digits being extended when the rat released the bar. In each session, 15–20 trials were performed and the 4 maximal forces (in grams) were averaged. Moreover, results were normalized by the weight of the animal (force/weight ratio).

The grip force independently exerted by each paw was measured with the same experimental procedure, except that the experimenter held the contralateral paw during the trial to ensure that the latter paw did not influence the result of the other paw. As previously mentioned, 15–20 trials were performed and the 4 maximal trials per session were averaged. The left forelimb force was then normalized by the right forelimb force (L/R ratio). Finally, each forelimb force was also normalized by the weight of the rat (ForceL/Weight ratio, for the left forepaw; ForceR/Weight ratio, for the right forepaw).

### 4. Electrophysiological measurements

#### Animal preparation

Rats were anesthetized by an intra-peritoneal injection of urethane solution (100 mg/100 g). Central temperature was maintained at about 38°C with a homeothermic blanket driven by a rectal thermal probe. Animals were positioned in dorsal *decubitus* during the surgery and all neuromuscular measurements. The shoulder and elbow were firmly held by clamps on a horizontal support to avoid disturbing movements and to maintain the 120° elbow joint angle during electrical nerve stimulations. The *pectoralis major* and *minor* muscles were removed and the left *musculospiral* nerve, innervating the *triceps brachii* muscle, was dissected free from surrounding tissues over a length of 3–4 cm. The other nerves of the brachial plexus were sectioned. Strain gauge was fixed at the wrist level and was opposed to the arm movement induced by nerve electrical stimulation.

#### Isometric exercise

**Figure 2:**
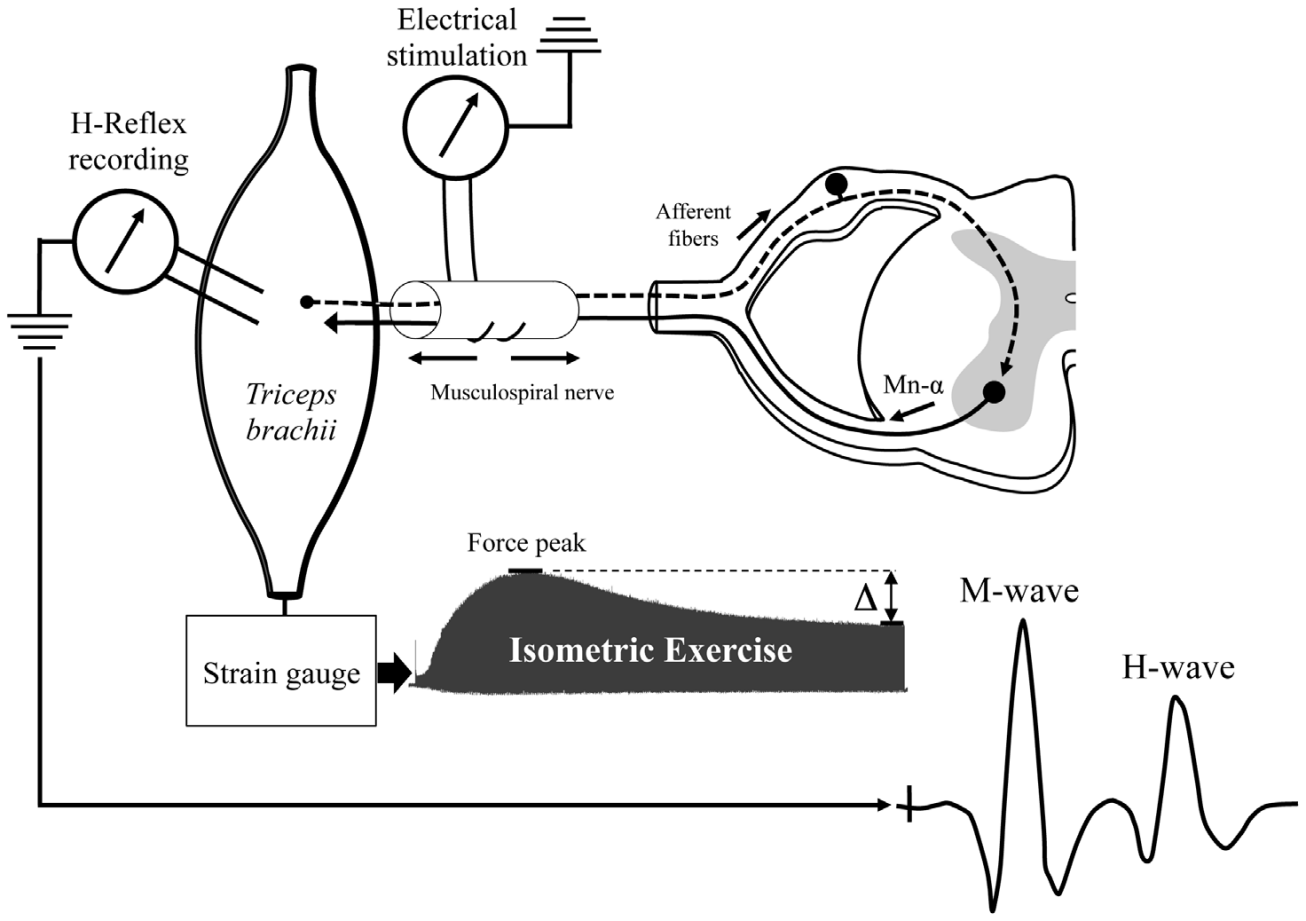
Schematic experimental procedure to evoke H-reflex and triceps brachii isometric exercise by musculospiral nerve stimulation.

The isometric exercise was characterized by a series of *triceps brachii* isometric contraction evoked by electrical *musculospiral* nerve stimulation (duration: 1 ms; frequency: 10 Hz). A constant current neurostimulator (Digitimer DS7 A; Welwin, Garden City, Hertforshire, UK) delivered single rectangular shocks to the *musculospiral* nerve using a pair of steel hooks. The current intensity used to evoke maximal twitch amplitude was measured from the beginning of the curve to the peak. The intensity of the isometric exercise was fixed to 25% of the twitch amplitude. To measure *triceps brachii* muscle isometric force, a wire connected to a strain gauge (Microdynamometer S 60; Houston, TX, USA) was perpendicularly fixed around the wrist. Contractions were recorded with Biopac MP150 system (sampled at 2,000 Hz, filtered with low pass at 150 Hz) and analyzed with Biopac AcqKnowledge 3.9 software (Goleta, USA). The isometric exercise was interrupted when the force fell to 50% of its peak value ***(*** ***Figure 2*** ***)***. The time of the exercise was measured.

#### H-reflex response before and after isometric exercise

The H-reflex was used to assess the spinal sensorimotor adaptation after MCAO-r by peripheral nerve stimulation. In our study, the *triceps brachii* H-reflex was evoked before and after an isometric exercise. To evoked the M and H-waves, single rectangular shocks were directly applied to the intact *musculospiral* nerve using a pair of steel hooks with a 1-msec pulse generated by a constant current stimulator. Stimulation electrodes were located at ∼4 mm of the nerve insertion into the muscle. In accordance with a previous study [*39*], the stimulation frequency was fixed at 0.1 Hz to abolish the post-activation depression during the consecutive H-reflexes recording. The H-reflex was recorded using bipolar needle electrodes (29-gauge; Oxford, UK; MLA 1204 needle electrodes, 2-mm pin) inserted into the belly of the *triceps brachii* muscle. It is noteworthy to add that the location of the electrodes was never changed throughout the H-reflex recordings (between the PRE-exercise and the POST-exercise measurements). The *triceps brachii* was selected because it seemed to be more appropriate than the other forelimb muscles. Being large and thick, it facilitates the location of EMG electrodes. Furthermore, because muscle length is known to influence H-reflex amplitude, all evoked potentials were recorded for a given elbow angle (i.e., 110°) [*40*]. The reflex signal was referred to a ground electrode implanted in an inert tissue, amplified (2k), and filtered (30 Hz to 10 kHz) with a differential amplifier (P2MP, 5104B, Marseille, France). The experimental design for H-reflex recording is shown in ***Figure 2***.

The stabilized maximal M-wave (M_max_) and H-wave (H_max_) peak-to-peak amplitudes were determined by incrementally increasing stimulation intensity (by 0.01-mA increments) from 0 mA until there was no further wave amplitude increase. Stimulation intensity was fixed to obtain H_max_ and M_max_. Then, five H-reflexes were evoked before and five other H-reflexes were elicited after the isometric exercise. The H_max_/M_max_ ratio was then calculated for each evoked H-reflex and averaged. Changes in H_max_/M_max_ ratio after exercise were expressed as percentage of the corresponding H_max_/M_max_ ratio before exercise.

### 5. Histology

#### Transcardiac perfusion

After electrophysiological recordings, the left cardiac ventricle was exposed and drilled. All animals were then perfused transcardially (25 ml/min) with 250–300 ml of 0.1 M ice-cold phosphate buffer (7.2<pH<7.4) and followed by the same volume of 4% phosphate-buffered paraformaldehyde (pH 7.4). Tissues were immediately dissected, post-fixed for 2 h at 4°C in the same fixative buffer and cryoprotected 24 h at 4°C in 30% sucrose. Tissues were then snap-frozen at −40°C for 10 s in Isopentane solution and stored at −80°C [*41*]. The next step consisted to perform coronal sections of rat brain (slide thickness: 30 µm).

#### Cresyl violet staining

Cresyl violet staining is commonly used method to quantify experimental brain infarctions. Sections were rinsed in distilled water for 5 min and incubated 3 min in a cresyl violet bath. Sections were then dehydrated through a sequence of ethanol baths (70, 95, and 100%). Sections were finally cleaned in Xylene during 2 min and medium mounted with coverslip using Permount (Fair Lawn,NJ, USA). For each section, the infarction area and total area of each hemisphere were delimitated and calculated with ImageJ software [*42*].

### 6. Magnetic Resonance Imagery (MRI)

Anesthesia was induced with 5% isoflurane and maintained with 2–2.5% isoflurane through a facemask, supplemented with oxygen. The MRI experiments were performed in a horizontal Bruker Biospec 4.7-T (Bruker Biospec Avance 4.7T/30, Bruker, Ettlingen, Germany). The core temperature was maintained close to 37°C by circulating warm water in a heating pad placed under the torso. Breathing rate was also monitored throughout the MRI study. The animal head was secured by ear and tooth bars to limit movement. Then, the surface coil was placed above the head for brain imaging. Two types of measurements were performed: 1) Twenty coronal slices were from T_2_ weighing (contiguous slices; TE: 50 ms; slice thickness: 1 mm; matrix: 256×256 pixels; field of view: 40×40 mm^2^). By the use of the ImageJ software, the regions of interest were selected manually. The edema volume was compared to the right hemisphere volume and to the total brain volume. 2) Twenty coronal slices were from T_2_* weighing (contiguous slices; TE: 15 ms; idem for the other parameters) to observe blood accumulation (intracerebral hemorrhages) [*43*].

### 7. Experimental protocol

In a first step, rats were familiarized with the elevated body swing test, the beam balance test, the ladder-climbing test and the forelimb grip force by training them 4 times a week during 1 week. Then these behavioral tests were realized once before the surgery (PRE) and daily after it from the 1^st^ day to the 7^th^ day (D1, D2, D3, D4, D5, D6 and D7). The H-reflex recordings before and after isometric exercise were performed at D1 for MCAO-D1 and SHAM-D1 groups, but also, at D7 for MCAO-D7 and SHAM-D7 groups. Finally after electrophysiological measurements, the size of cerebral infarction was measured by histological analysis with Cresyl violet staining. To be more complete, the size of cerebral edema was measured by MRI on 1 Control rat, 1 SHAM rat and 2 MCAO-r rats at D1 and D7.

### 8. Statistical analysis

Statistical analysis was performed using SigmaStat software program (Statistical software, San Jose, CA, USA). All data are presented as Mean±SD and where compared by ANOVA tests. Post-hoc comparisons were performed with Student-Newman-Keuls multiple post-test comparisons. Results were considered statistically significant, highly significant or very highly significant if the p-value fell below 0.05, 0.01 and 0.001, respectively.

## Results

The rat weight decreased between 11% and 19% after MCAO-r throughout protocol duration while the weight of SHAM rats was similar to the one of Control rats.

No difference was observed from PRE to D7 in Control and SHAM-D7 groups for all the measured behavioral parameters, except for grip force (g) in SHAM-D7 (see below: 4.). Likewise, no difference was observed between these 2 groups.

Results of the behavioral assessment obtained in MCAO-D1 group were similar to the ones of the MCAO-D7 group at D1 (MCAO-D1 group results, see ***Table 1***). No difference was observed between Control and SHAM-D1 groups.

**Table 1:** Behavioral tests for Control, SHAM-D1 and MCAO-D1 groups.

	Control		SHAM-D1		MCAO-D1	
	PRE	D1	PRE	D1	PRE	D1
**Elevated body swing test**	0.5±0.2	0.5±0.2	0.52±0.13	0.58±0.13	0.5±0.13	1^+/*^
**Ladder-climbing test**	0.93±0.05	0.96±0.03	0.94±0.05	0.91±0.04	0.94±0.02	0.43±0.18^+/*^
**Beam balance test**	120	120	120	120	120	0^+/*^
**Force with 2 the forepaws (g)**	853±87	930±124	939±85	967±135	943±105	883±99
**Force/Weight ratio**	3.5±0.2	3.6±0.3	3.6±0.3	3.7±0.3	3.7±0.4	4.0±0.5^+/*^
**Force L/R ratio**	0.92±0.09	1.00±0.14	0.96±0.09	0.98±0.12	1.09±0.08	0.69±0.07^+/*^

The PRE and D1 values are represented. ^*^ indicates significant differences between PRE and D1 (p<0.001). ^+^ indicates significant differences between MCAO-D1 group and the 2 other groups (p<0.001).

### 1. Elevated body swing test (EBST)

**Figure 3:**
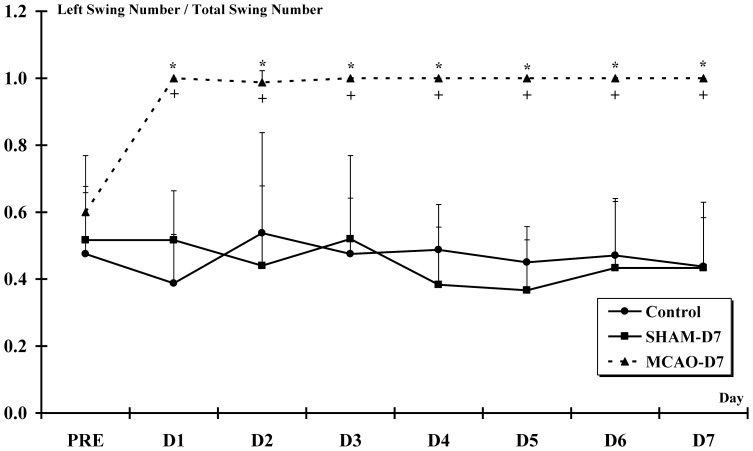
The elevated body swing test. This test is used to assess the lateralization of the lesion (asymmetrical motor behavior). * indicates a significant increase for the left swing number/total swing number ratio for MCAO-D7 group between PRE and from D1 to D7 (p<0.001). + indicates a significant increase for the left swing number/total swing number ratio for MCAO-D7 group compared to Control and SHAM-D7 groups from D1 to D7 (p<0.001).

The animals exclusively straightened up on the left side during the 7 days post-MCAO-r. Indeed, the left swing number/total swing number was significantly higher from D1 to D7 compared to PRE in MCAO-D7 group (PRE = 0.6±0.17; from D1 to D7 = 1; p<0.001). ***(*** ***Figure 3*** ***)***. The left swing number/total swing number observed in the MCAO-D7 group was significantly higher than Control and SHAM-D7 groups from D1 to D7 (p<0.001).

### 2. Beam balance test

**Figure 4:**
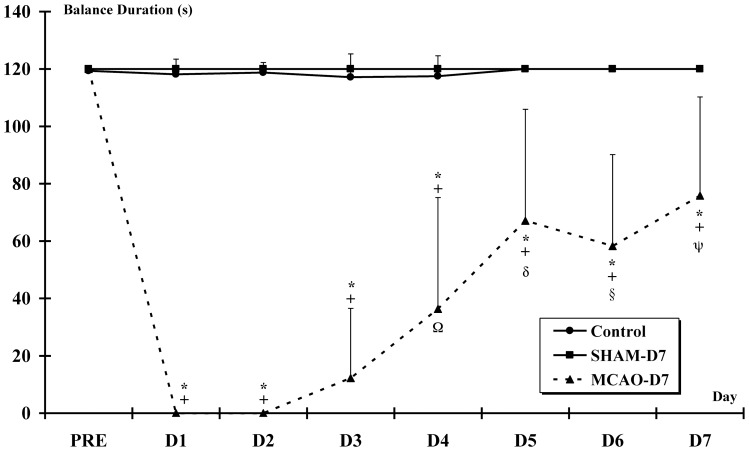
Beam balance test. The beam balance task is used to assess deficits of the vestibulomotor function after MCAOr. * indicates a significant decrease in the balance duration for MCAO-D7 group between PRE and from D1 to D7 (p<0.001). + indicates a significant decrease in the balance duration for MCAO-D7 group compared to Control and SHAM-D7 groups from D1 to D7 (p<0.001). For the MCAO-D7 group: Ω indicates a significant difference between D4 and D1 to D3; δ indicates a significant difference between D5 and D1 to D4; § indicates a significant difference between D6 and D1 to D4 (p<0,001 for all); ψ indicates a significant difference between D7 and D1 to D4 (p<0.001 for all).

The balance deficits persisted after 7 days post-MCAO-r. The balance time on the rod in the MCAO-D7 group from D1 to D7 was strongly inferior to PRE values (p<0.001) ***(*** ***Figure 4*** ***)***. However, the balance time was significantly higher from D4 to D7 (36±39 s; 67±39 s; 58±32 s; 76±34 s respectively) compared to D1, D2 and D3 (p<0.001). The balance time was significantly shorter from D1 to D7 in MCAO-D7 than Control and SHAM-D7 groups (p<0.001).

### 3. Ladder-climbing test

**Figure 5:**
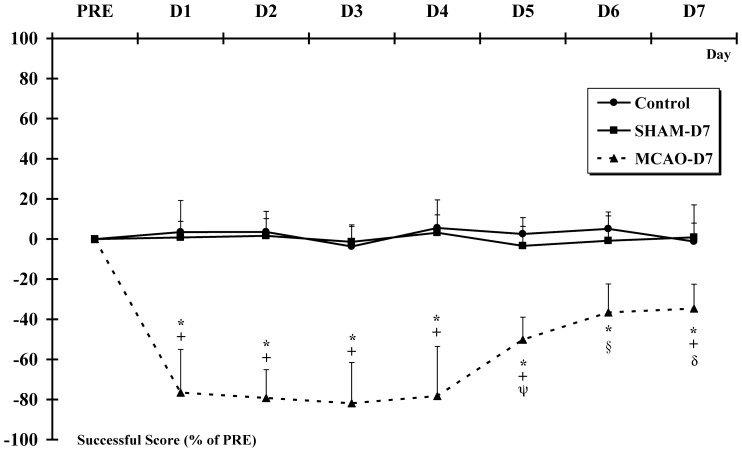
The ladder-climbing test. The ladder-climbing test is used to evaluate the sensorimotor capacities to correctly grip the rung while rats climbed up a inclined ladder. * indicates a decrease of the successful score in the MCAO-D7 group between PRE and D1 to D7 (p<0.001). The successful score is expressed in % in the graph. **+** indicates a decrease of the successful score in the MCAO-D7 group compared to Control and SHAM-D7 groups from D1 to D7 (p<0.001). For the MCAO-D7 group: δ indicates a significant difference between D7 and D1 to D5; **§** indicates a significant difference between D6 and D1 to D5; ψ indicates a significant difference between D5 and D1 to D4 (p<0.001).

The animals showed strong difficulties to climb and grip the ladder with the left paw during 7 days post-MCAO-r. Indeed, the successful score significantly decreased from D1 to D7 in MCAO-D7 group compared to PRE (p<0.001) ***(*** ***Figure 5*** ***)***. Moreover, the successful score was significantly higher from D5 to D7 (0.44±0.09; 0.57±0.14; 0.59±0.12 respectively) compared to D1-D4 (0.21±0.20; 0.19±0.13; 0.16±0.18; 0.20±0.22 respectively; p<0.001 for all values). The successful score was significantly lower in MCAO-D7 group from D1 to D7 than Control and SHAM-D7 groups (p<0.001).

### 4. Forelimb grip force

The force decreased after MCAO-r but increased when the force was normalized to the body weight.

#### Changes in grip force exerted by both forepaws throughout protocol

**Figure 6:**
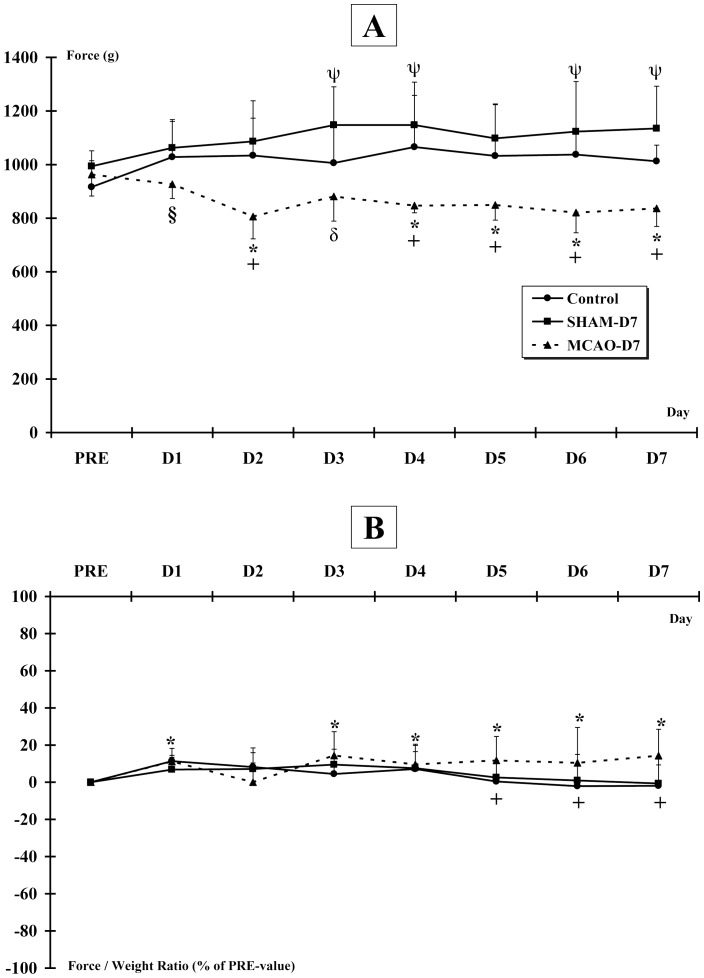
Grip force exerted by both forepaws. The grip force is measured by using a grip force tester. **A**. Absolute grip force (g). * indicates a decrease of force for the MCAO-D7 group between PRE and D2, D4, D5, D6, D7. § indicates a decrease of force between D1 and D2 for the MCAO-D7 group. + indicates a significant difference between MCAO-D7 group and Control and SHAM-D7 groups (p<0,01).δ indicates a significant difference between MCAO-D7 group and SHAM-D7 group. ψ indicates an increase of force for the SHAM-D7 group at D3, D4, D6 and D7. **B**. Relative grip force (force/weight ratio, % of PRE-value). * indicates a decrease of force for the MCAO-D7 group between PRE and D1, D3, D4, D5, D6, D7 (p<0.05). + indicates a significant difference between MCAO-D7 group and Control (p<0.01 at D5, D6 and p<0.001 at D7) and between MCAO-D7 and SHAM-D7 groups (p<0.05 at D5, D6 and p<0.001 at D7).

The force (g) significantly increased in SHAM-D7 group at D3, D4, D6 and D7 (1148±143 g; 1148±160 g; 1123±186 g; 1135±158 g respectively, p<0.05) compared to PRE (994±58 g). However, when the force was normalized by the rat's weight (force/weight), no difference was observed in SHAM-D7 group during the protocol, as in Control group. A decrease of force for the MCAO-D7 group was noticed between PRE and D2, D4, D5, D6, D7 whereas no difference was observed at D1 and D3 (927±53 g; 881±92 g respectively, p = 0.07)***(*** ***Figure 6A*** ***)***. However, the force/weight ratio was increased in MCAO-D7 group at D1, D3, D4, D5, D6 and D7 (927±53 g; 881±92 g; 847±27 g; 849±57 g; 821±75 g; 837±67 g respectively; p<0.05) compared to PRE (963±80 g)***(*** ***Figure 6B*** ***)***. Moreover, the observed force at D1 in MCAO-D7 group was significantly higher than the force at D2 (p<0.05).

#### Changes in grip force exerted by both forepaws between groups

The force in MCAO-D7 group was significantly lower from D2 to D7 than Control (p<0.01) and SHAM-D7 (p<0.001) groups, except at D3 where the force in MCAO-D7 group was only lower than SHAM-D7 group (p<0.001)***(*** ***Figure 6A*** ***)***. The force/weight ratio for MCAO-D7 group was higher at D5, D6 and D7 than the force/weight ratio for Control (p<0.01, p<0.01 and p<0.001 respectively) and for SHAM-D7 groups (p<0.05, p<0.05 and p<0.01 respectively)***(*** ***Figure 6B*** ***)***.

#### Changes in grip force exerted by each forepaws

As described below, the modified grip force production was associated with a force decline of the left forelimb and an increase of right forelimb force.

#### The L/R ratio (Left/Right)

**Figure 7:**
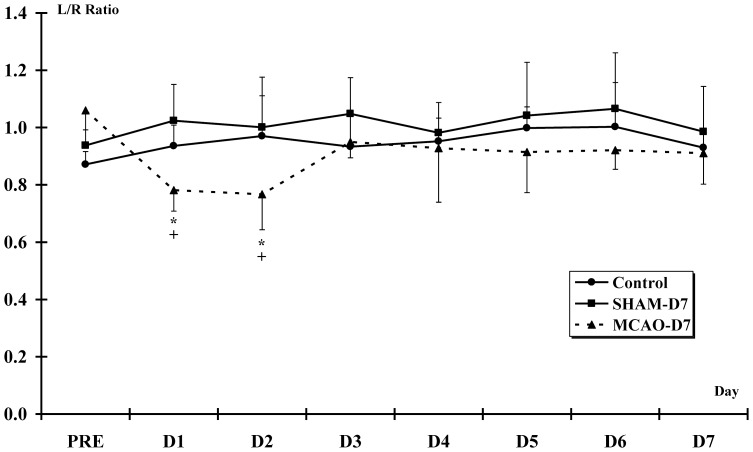
Grip force exerted by each forepaw - the L/R ratio. * indicates a decrease of L/R ratio for the MCAO-D7 group between PRE and D1-D2. + indicates a significant difference for the MCAO-D7 group and the Control and SHAM-D7 groups (p<0.01).

The PRE L/R ratio of the MCAO-D7 group significantly decreased compared to the D1 and D2 L/R ratio (PRE: 1.1±0.1; D1 and D2: 0.8±0.1; p<0.001 for both) ***(*** ***Figure 7*** ***)***. The L/R ratio in the MCAO-D7 group was lower than in the Control at D1 and D2 (PRE: 0.9±0.04; D1: 0.9±0.1; D2: 1.0±0.1; p<0.01 for both) and in the SHAM-D7 (PRE: 0.9±0.1; D1: 1.0±0.1; D2: 1.0±0.2; p<0.001 for both) groups.

#### The left forepaw force

The force in the MCAO-D7 group was significantly lower from D1 to D7 (358±36 g; 344±54 g; 356±57 g; 342±81 g for D1, D2, D6 and D7 respectively; p<0.001; 419±32 g; 387±55 g; 390±41 g for D3, D4 and D5 respectively; p<0.05) compared to PRE (475±51 g). The ForceL/Weight ratio significantly decreased at D1 (p<0.01) and D2 (p<0.001) compared to D3 in MCAO-D7 group (*data not shown*). The force (g) in MCAO-D7 group was significantly lower than Control and SHAM-D7 groups from D1 to D7 (p<0.05), except for D3 (*data not shown*).

#### The right forepaw force

Contrary to the force decline observed at D7 compared to PRE (450±38 g, p<0.05), D1 (458±20 g; p<0.01), D2 (451±51 g; p<0.05), D3 (442±40 g; p<0.05) and D5 (436±80 g; p<0.05), no difference was observed from D1 to D6 compared to PRE in MCAO-D7 group. However, the forceR/Weight ratio was significantly increased from D1 to D5 in MCAO-D7 group compared to PRE (p<0.01 for both) (*data not shown*). The force was significantly lower in MCAO-D7 group compared to Control at D7 (p<0.01) and to SHAM-D7 group at D6 and D7 (p<0.01). However, the forceR/Weight ratio in the MCAO-D7 group was significantly higher than the Control and SHAM-D7 groups from D2 to D7 (for D4 and D7, p<0.05; for D6, p<0.01; for D2, D3 and D5, p<0.01). At D1, the forceR/Weight ratio in the MCAO-D7 group was significantly higher than the Control group (p<0.05) (*data not shown*).

### 5. Electrophysiology: H-reflex response before and after isometric exercise

The right MCAO-r induced a change in the motor reflex regulation after isometric exercise of the contralateral triceps brachii muscle one day, but not seven days after the cerebral infarction. It was also found that there was an earlier muscle fatigue during exercise after such injury.

#### Isometric exercise

**Figure 8:**
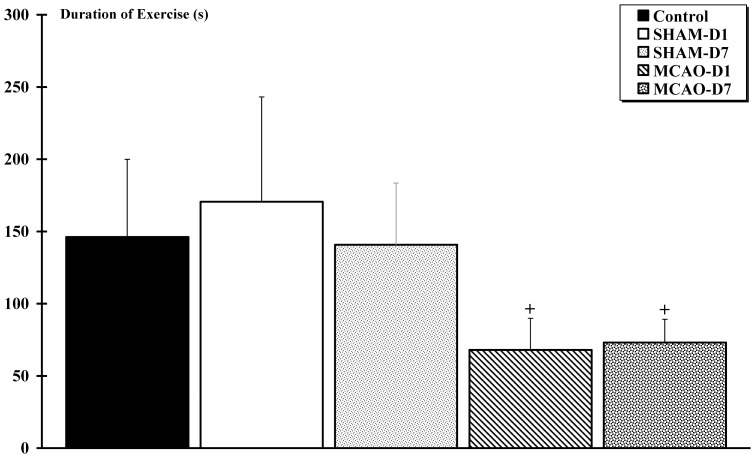
Duration of isometric exercise evoked by electrical stimulation of the musculospiral nerve. + indicates a significant difference between MCAO-D1 and MCAO-D7 groups compared to Control, SHAM-D1and SHAM-D7 groups (p<0.01).

The duration of exercise was decreased after 2-hour MCAO-r from the first day until the end of the protocol. The times of isometric exercise to reach -50% of the peak value in MCAO-D1 (68±22 s) and MCAO-D7 (73±16 s) groups were significantly shorter (p<0.01) than SHAM-D1 (171±73 s), SHAM-D7 (141±43 s) and Control (146±54 s) groups. No difference was observed between SHAM-D1, SHAM-D7 and Control ***(*** ***Figure 8*** ***)***.

#### H-reflex before and after isometric exercise

**Figure 9:**
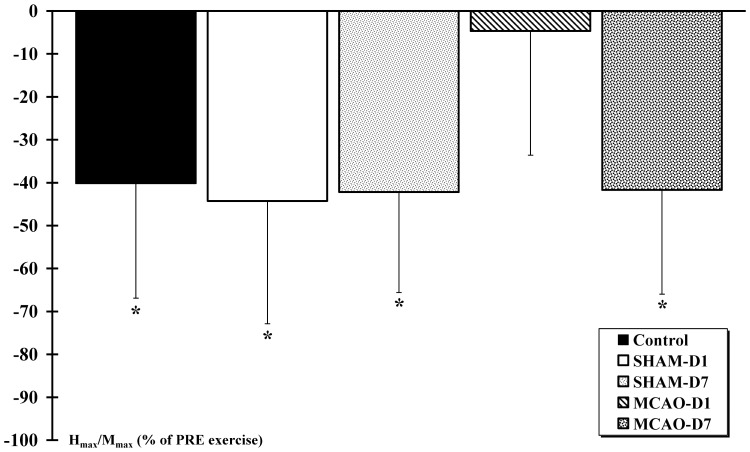
H_max_/M_max_ ratios after isometric exercise. The M- and H-waves are evoked by stimulation of the musculospiral nerve. * indicates a significant decrease of H_max_/M_max_ evoked after exercise compared to the H_max_/M_max_ evoked before exercise for the Control, SHAM-D1, SHAM-D7 and MCAO-D7 groups (p<0.01). No difference was observed for the MCAO-D1 group.

The measured H-reflex in resting condition was not modified after ischemia. However, the H-reflex response to isometric exercise was altered one day after lesion and was recovered 7 days after the lesion. Indeed, the H_max_/M_max_ ratio after isometric exercise decreased significantly in Control (−40±27%), SHAM-D1 (−44±28%), SHAM-D7 (−42±23%) and MCAO-D7 (−42±24%) compared to the H_max_/M_max_ before exercise (p<0.01 for all groups), but not for the MCAO-D1 group in which the H_max_/M_max_ ratio did not significantly differ after exercise (−5±29%)(***Figure 9***). No difference was observed in the H_max_/M_max_ ratio between groups before and after exercise.

### 6. Histology: Cresyl violet staining

**Figure 10:**
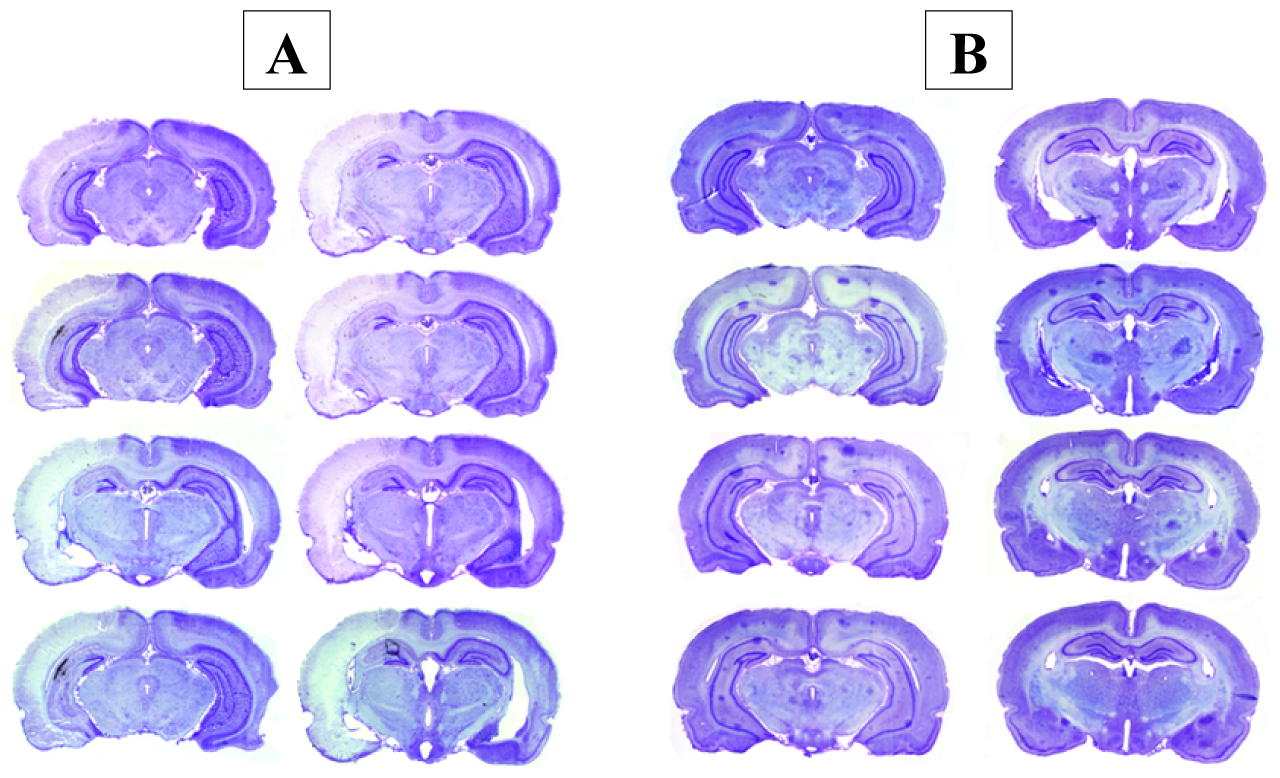
Representative coronal slices with Cresyl violet following MCAO-r. **A**. Slices recorded at MCAO-D1. **B**. Slices recorded at SHAM-D1.

**Figure 11:**
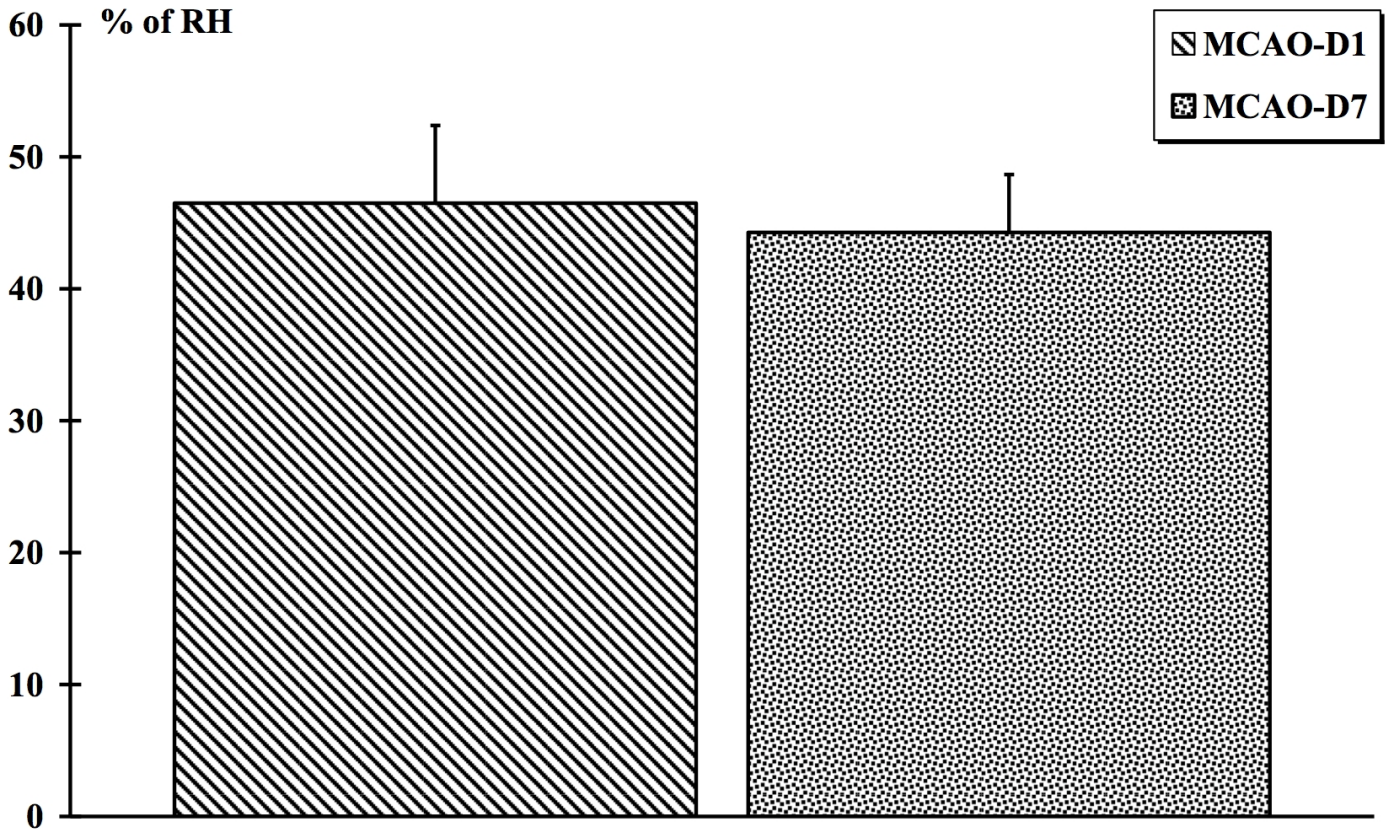
Infarct volume measurement from the Cresyl violet staining. The cerebral lesion size observed in the right hemisphere (RH) after MCAO-r was measured at D1 and D7. Values are expressed in % of the RH.

The infarction size for the MCAO-D1 group was spread over half of the right hemisphere ***(Figure 10.A)***. The lesion size tended to be slightly decreased in the MCAO-D7 group compared to the MCAO-D1 group ***(*** ***Figure 11*** ***)***. However, no lesion appeared in the SHAM ***(Figure 10.B)*** and Control animals.

**Figure 10. Representative coronal slices with Cresyl violet following MCAO-r.** **A**. Slices recorded at MCAO-D1. **B**. Slices recorded at SHAM-D1.

### 7. MRI: Size of cerebral edema

**Figure 12:**
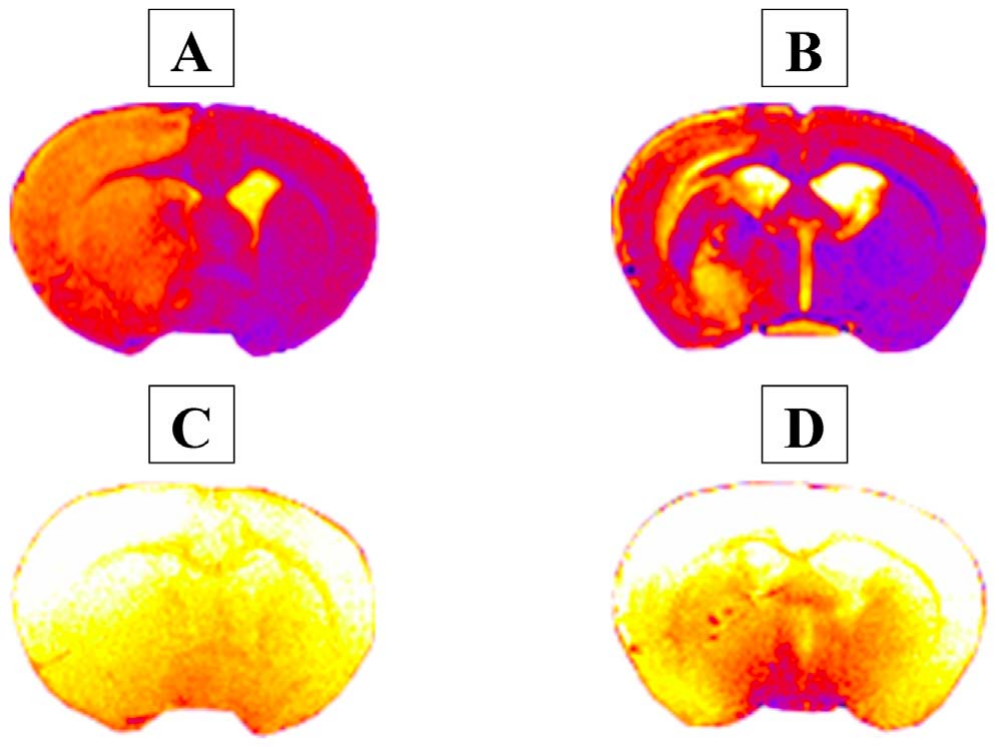
MRI following MCAO-r on 2 rats. Representative coronal slices obtain with T_2_-weighting: **A**. at D1 **B**. at D7 post-ischemia. Representative coronal slices obtain with T_2_* weighting: **C**. at D1, **D**. at D7 post-ischemia.

MRI analysis showed no edema and no cerebral hemorrhages in Control and SHAM groups. Concerning the 2 MCAO animals, cerebral edema was observed at the right hemisphere without affecting the left one from T_2_ weighting ***(*** ***Figure 12*** ***A/B)***. For the first rat, edema covered 51% of the right hemisphere and 27% of total brain at D1. The edema volume showed a 29% decline at D7 when compared to the right hemisphere volume, and, a 30% decline when compared to the total brain volume. For the second rat, the edema volume was similar to the first rat at D1 and D7 ***(*** ***Table 2*** ***)***. Moreover, no cerebral hemorrhage was observed for these rats from T_2_* slices ***(*** ***Figure 12*** ***C/D)***. No sign of lesion appeared in the SHAM and Control animals.

**Table 2:** MRI analysis (T_2_).

	Rat 1		Rat 2	
	D1	D7	D1	D7
**Edema Volume (mm^3^)**	447	284	450	302
**Right Hemisphere Volume (mm^3^)**	868	789	871	792
**Brain Volume (mm^3^)**	1689	1565	1656	1554

Size of the cerebral edema at the right hemisphere after MCAO-r on 2 rats. The measures are realized at D1 and D7.

## Discussion

The main results of the present study indicated that the right MCAO-r induced a change in the motor reflex regulation after isometric exercise of the contralateral *triceps brachii* muscle. The rats that underwent a 2-hour cerebral ischemia displayed an earlier muscle fatigue during isometric exercise throughout the protocol. In addition, we deepened the functional deficits level induced by cerebral ischemia through the analysis of behavioral tests assessing force production, static balance and sensorimotor alterations.

### 1. Methodological considerations

The similar results obtained in Control and SHAM groups confirmed that the MCAO-r surgery without cerebral ischemia did not influence the recorded neuromuscular parameters. Furthermore, the results of different behavioral tests for Control and SHAM groups were not modified throughout the protocol meaning that these measurements were reliable during one week. However, the increased grip force of both forelimbs in the SHAM group during the first week post-surgery could be associated with stress often present after surgical procedure [*44*]. Nevertheless, this result stayed opposed to the one observed in cerebral lesioned animals. The observed functional deficits could thus be related with the MCAO-r effects in our study.

Looking at the infarct volume (Cresyl violet staining), the MCAO-r reliably affected the right hemisphere and thus induced a reproducible lesion. In addition, the histological analysis by Cresyl violet staining indicated that the infarct volume showed a tendency to decrease between D1 and D7 confirming previous studies [*28*], [31], [*45*]. Although MRI was performed on few rats, cerebral edema clearly showed an increase one day after MCAO-r and decreased at the 7^th^ day that was in accordance with previous studies [*25*], [*27*]. The infarct volume could be explained by the fact that the edema exacerbated the lesion size. Being maximal at one day post-ischemia, the edema accentuated the lesion size while decreasing it at the end of the first week. We could suggest that the spinal sensorimotor adaptation was assessed on animals with a similar cerebral lesion in our study.

Several methodological criteria were respected to evoke *triceps brachii* H-reflex and ensured that the recording conditions of H-reflex were similar for each animal in our study: 1) H-wave progressively increased from the first deflection to the maximal H-wave amplitude when the stimulation intensity was incremented, as previously described [*46*], 2) the stimulation intensity to elicit H_max_ was higher than or equal to the stimulation intensity necessary to elicit M_max_ [*47*], 3) H-reflex was not abolished when electrical stimulation was applied at M_max_ intensity, 4) M-wave amplitude was always higher than H-wave regardless of the stimulation intensity and 5) the latency between M- and H-waves was similar during the different recordings for each animal. It seems important to notice that the location of recording intramuscular electrodes was not modified during the experiments to ensure a reliable comparison between the PRE- and POST-exercise recordings. Moreover, the H_max_/M_max_ ratio limited the influence of muscle membrane excitability that could change the interpretation of H-reflex results [*40*].

### 2. The motor reflex response to exercise is disturbed after MCAO-r

The H-reflex, obtained before exercise, was not modified by cerebral ischemia. This result was in agreement with a previous study on humans in which no difference of the *extensor carpi radialis longus* and *flexor carpi radialis* H-reflex in resting condition was detected after stroke injury [*19*]. Other study showed an opposite result by measuring an increase in H-reflex after stroke injury [*18*]. Such controversial observation between these two studies may be explained by different methodological approach for measuring H-reflex (H_max_ amplitude vs. H_slope_/M_slope_ and H_max_/M_max_). It suggests that the synaptic transmission between large diameter afferent fibers (group I and II) and α-motoneurons seems not to be altered following MCAO-r.

However, the H-reflex response to exercise was changed one day after lesion. Indeed, contrary to the H-reflex response of the reference groups (Control and SHAM) that strongly decreased after isometric exercise, the motor reflex response of the injured rats remained stable after exhaustive exercise. To explain this result, we suggested that the neural network activated during exercise and regulating the H-reflex response in the spinal cord might be modified after MCAO-r. Indeed, the regulation of the H-reflex amplitude is associated with the activity of motor descending pathways and with the afferents of the active muscle. It was also found that the afferents from antagonist and/or synergic muscles and cutaneous/articular afferents could change the H-reflex amplitude [*22*]. In our study, the nerves of the synergic and antagonist muscles of the *triceps brachii* were sectioned to avoid the influence of their afferent fibers during H-reflex recordings. Consequently, the mechanisms involving the surrounding muscles of the *triceps brachii* did not contribute to adjust the motor reflex activity after the isometric exercise.

Given that the isometric exercise was exhaustive, the active muscle may accumulate metabolite known to activate the muscle afferents from groups I, II (mechanosensitive), III and IV (mechano- and metabosensitive) [*23*], [*48*]. In addition, cerebral damages may disturb descending pathways regulating motor response at the spinal reflex [*3*]. Consequently, the H-reflex response to exercise might be associated with changes of descending pathway activity on *triceps brachii* motoneurons and/or changes of the *triceps brachii* muscle afferent activity. Neural mechanisms underlying H reflex response to exercise remains to determine in further studies.

### 3. The H-reflex response to exercise seemed to recover at the end of the first week post-cerebral ischemia

The H-reflex response to isometric exercise was normalized as soon as the first week post-MCAO-r. Indeed, the H_max_/M_max_ ratio decreased after exercise one-week post-MCAO-r as in the reference groups. It means that the mechanisms underlying the motor reflex regulation evolved during the first week post-ischemia. The interpretation of such result needs caution because we could not affirm that the regulation of motor reflex returned to the same pre-injury state. The decrease of H-reflex after exercise could be associated with other neural mechanisms compared to control animals resulting in a similar H-reflex profile. Further studies are needed to clarify such neural strategy.

### 4. MCAO-r induced more important muscle fatigue from the first day

Our results confirmed that a disturbed motor control was associated with greater muscle fatigability after focal cerebral ischemia [*49*]. The well-known muscle atrophy and myotypology changes could not be responsible of such result the one-day after occlusion [*50*]. However, the observed fatigue could be partially explained by the modification of motor reflex activity affecting the motoneuronal recruitment. Indeed, it was demonstrated that force decline induced by exhaustive exercise could, to a certain degree, be explained by modification of the motor reflex response [*51*], [*52*]. Nevertheless, several authors demonstrated that one week after 1-hour cerebral ischemia induced a loss of muscle mass that affected both type II and type I muscle fibers [*50*]. The isometric exercise-induced fatigue, observed 7 days after MCAO-r, may be related to muscle atrophy and/or myotypology changes.

### 5. Functional deficits were maintained during the acute phase after MCAO-r

The elevated body swing test results indicated for the first time that 2-hour cerebral ischemia induced a strong motor asymmetry without any signs of recovery. Previous studies shown that a 1-hour cerebral occlusion induced a preferential and stable left orientation in 85% of trials (from 1 day to 2 months post MCAO-r) [*36*], [*53*]. After a 30 min focal ischemia, the orientation of swing stayed on the left side but could be inferior to 75% of trials [*54*]. Therefore, our study suggests that the duration of MCAO influences the severity of motor asymmetry.

In our study, the recovery of the ability to climb and grip the ladder stayed incomplete at the end of the protocol. It was in accordance to a previous study that demonstrated an increase in foot fault (53.4%) following a 2-hour occlusion [*10*]. Several reasons might explain this result such as a deficit in propulsion, inter-limb coordination and an alteration of proprioception of the left forelimb muscles [*38*]. However, the respective contribution of these different mechanisms stayed difficult to quantify and should thus be further clarified.

Animals were not able to maintain balance on the wooden beams during the first 2 days following the MCAO-r suggesting vestibulomotor deficits. Moreover, we observed that rats were systematically oriented on the left side inducing an important loss of balance. Therefore, the motor asymmetry may contribute to affect static balance on beams. Our results allowed us to observe a progressive (incomplete) recovery of balance in static position from the 4^th^ day post-ischemia. Several studies considered that the beam balance test was not sensitive enough to detect changes in balance after cerebral ischemia [*10*], [*37*]. However, we performed a simple methodological modification for this test. We left a 5 cm space between the 2 wooden beams in order to force rats only to use their paws and not their belly that could strongly disturb balance results. Our methodological modification of the beam balance test may be considered in further studies to optimize the assessment of functional disorders.

A loss of force exerted by both forelimbs has already been observed in few studies after MCAO-r, but some controversial results remain [*14*], [55], [*56*]. Therefore, the force measurement was deepened in the present study. Our results indicated that the loss of weight resulting from MCAO-r seemed to play an important role for the force production. Indeed, we observed that the force exerted by both forelimbs showed an opposite pattern that is an increase when the force was normalized to the rat weight. Thus, we suggest that the weight should be systematically taken into account for force measurements.

Interestingly, the force exerted by the left forelimb showed opposite response to the one produced by the right forelimb. The force of the left forelimb showed a light decrease when the weight of the animal was taken into account. The absence of a substantial loss of force (contrary to human outcomes) may be explained by the fact that the MCAO-r did not directly affect the motor cortex area, but mainly disturbed cerebral structures, such as the striatum or the thalamus, involved in the regulation of motor activity [*11*], [*57*]. In parallel, the force produced by the right forelimb (normalized by the weight) significantly increased during protocol. The compensation observed at the right forelimb level might represent a major force adaptation following transient focal cerebral ischemia in rats. The motor asymmetry, revealed this time by the L/R ratio, only lasted a couple of days and may be linked to the short-lasting decrement of left forelimb force.

In addition, it is noteworthy to add that the animals showed difficulties to grip the bar with the left forepaw when using it separately to the right paw. However, such difficulties were not observed when animals used both left and right forepaws. It might mean that the sum of the force exerted separately by each forelimb partially reflected the force produced by forelimbs together. It could be suggested with caution that the increment of force exerted by both forelimbs (when normalized by the weight) was associated with the increase of the right forelimb force.

## Conclusion and Clinical Perspectives

To the best of our knowledge, the present study was the first to show an alteration of the motor reflex regulation following MCAO-r. The neuromuscular adaptations in the acute and chronic phases should be more investigated, as they are essential to understand the neural modulations following severe cerebral ischemia [*3*]. Consequently, the assessment of treatments should be optimized and more complete. Our study also indicated that the MCAO-r method allowed clarifying the neuromuscular adaptations in the acute phase.

In addition, this study confirmed that the use of the physical exercise as a diagnostic tool could reveal neuromuscular changes that cannot be found in resting condition. Indeed, the alteration of the motor reflex regulation could not be detected without the use of the isometric exercise during electrophysiological recordings. The exercise as a model should be more taken into account in further studies.
